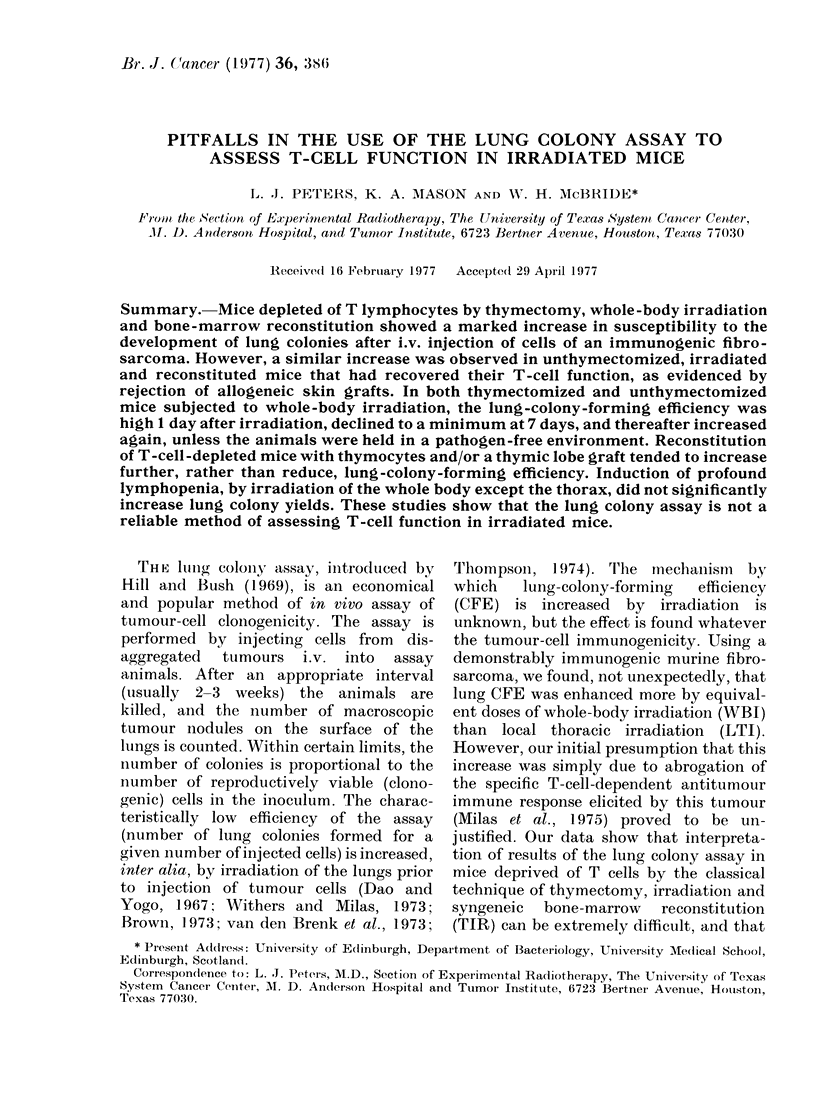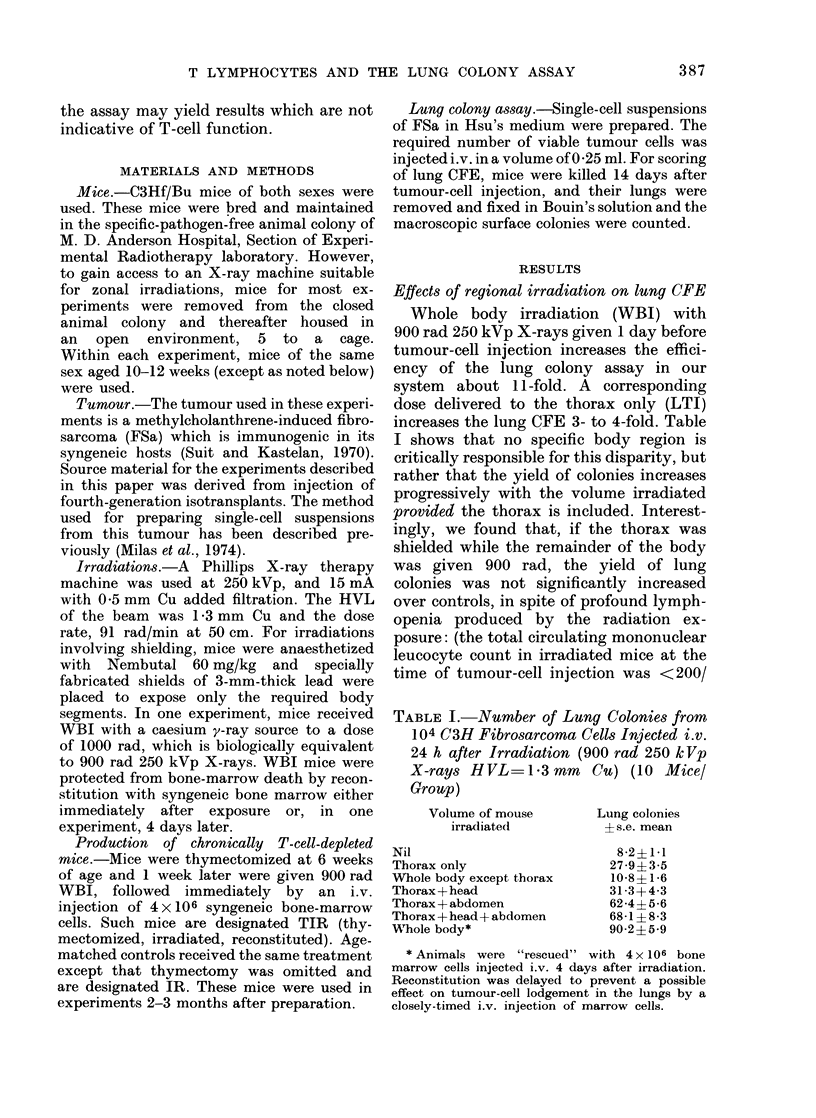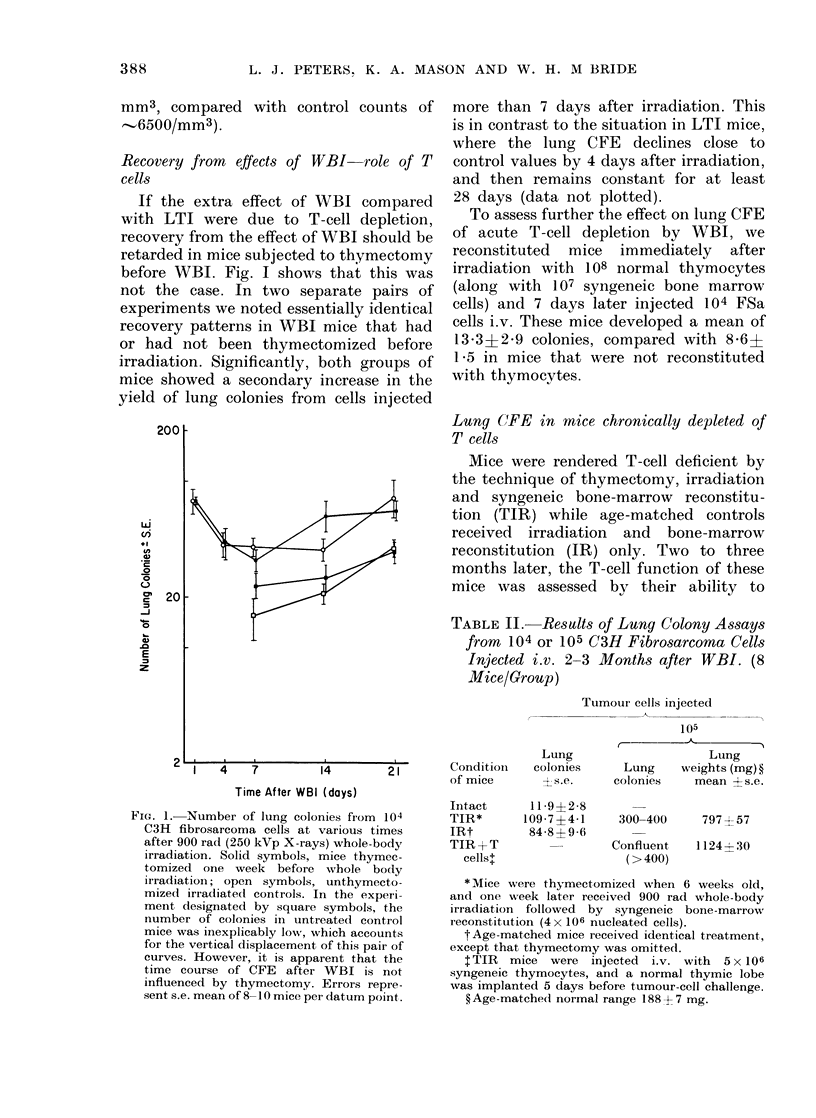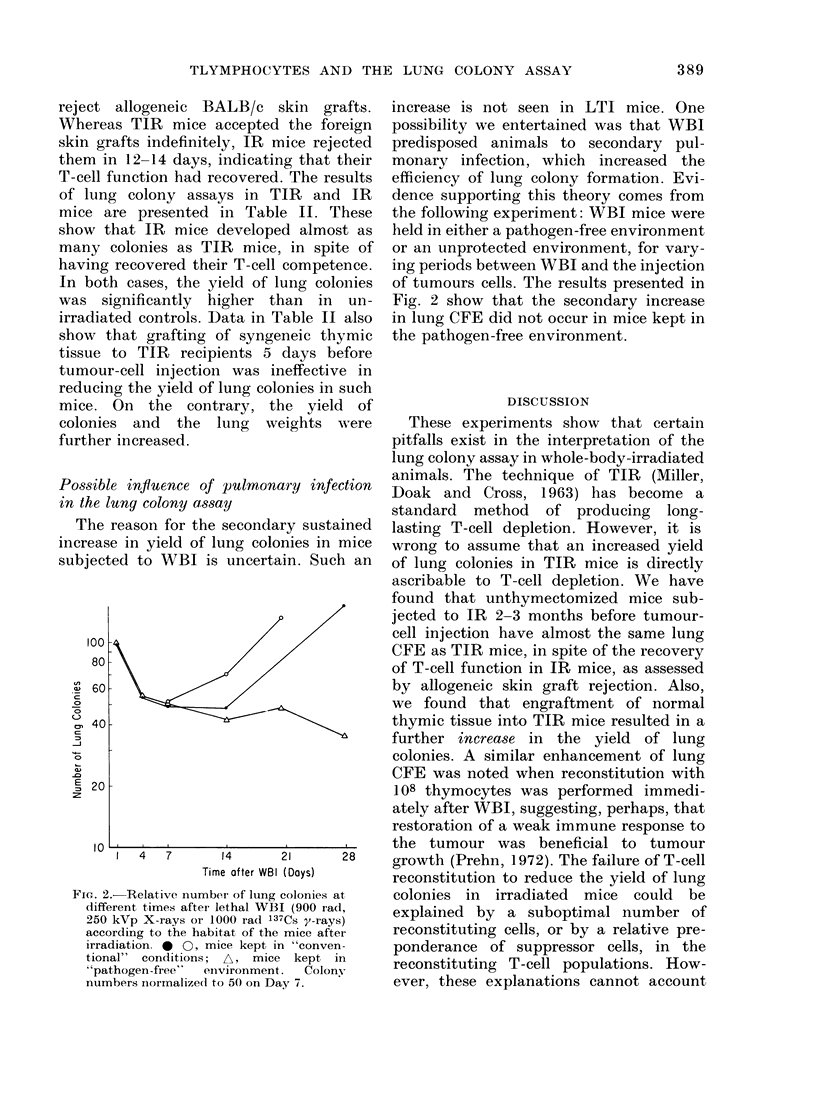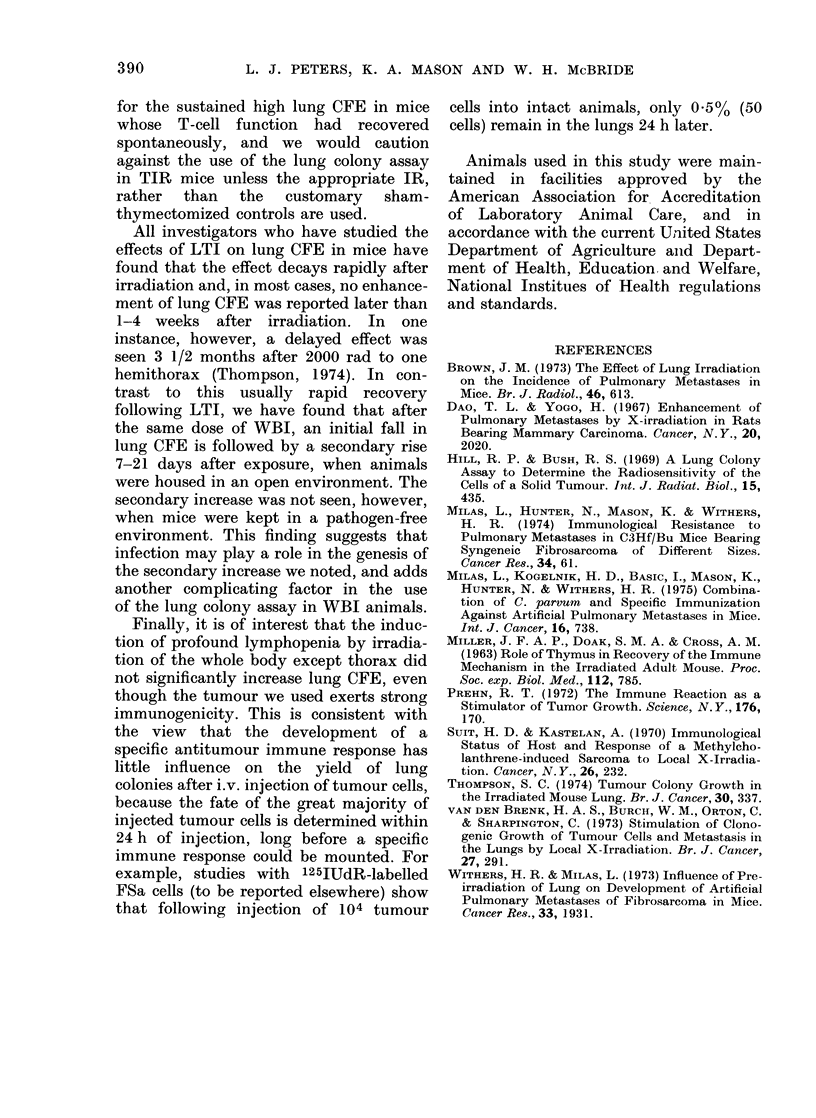# Pitfalls in the use of the lung colony assay to assess T-cell function in irradiated mice.

**DOI:** 10.1038/bjc.1977.204

**Published:** 1977-09

**Authors:** L. J. Peters, K. A. Mason, W. H. McBride

## Abstract

Mice depleted of T lymphocytes by thymectomy, whole-body irradiation and bone-marrow reconstitution showed a marked increase in susceptibility to the development of lung colonies after i.v. injection of cells of an immunogenic fibrosarcoma. However, a similar increase was observed in unthymectomized, irradiated and reconstituted mice that had recovered their T-cell function, as evidenced by rejection of allogeneic skin grafts. In both thymectomized and unthymectomized mice subjected to whole-body irradiation, the lung-colony-forming efficiency was high 1 day after irradiation, declined to a minimum at 7 days, and thereafter increased again, unless the animals were held in a pathogen-free environment. Reconstitution of T-cell-depleted mice with thymocytes and/or a thymic lobe graft tended to increase further, rather than reduce, lung-colony-forming efficiency. Induction of profound lymphopenia, by irradiation of the whole body except the thorax, did not significantly increase lung colony yields. These studies show that the lung colony assay is not a reliable method of assessing T-cell function in irradiated mice.


*Br. J. Cancer* (1977) **36**, 386

### PITFALLS IN THE USE OF THE LUNG COLONY ASSAY TO ASSESS T-CELL FUNCTION IN IRRADIATED MICE

L. J. PETERS, K. A. MASON AND W. H. McBRIDE*

*From the Section of Experimental Radiotherapy, The University of Texas System Cancer Center, M. D. Anderson Hospital, and Tumor Institute*, 6723 *Bertner Avenue, Houston, Texas* 77030

Received 16 February 1977 Accepted 29 April 1977

**Summary.—Mice depleted of T lymphocytes by thymectomy, whole-body irradiation and bone-marrow reconstitution showed a marked increase in susceptibility to the development of lung colonies after i.v. injection of cells of an immunogenic fibrosarcoma. However, a similar increase was observed in unthymectomized, irradiated and reconstituted mice that had recovered their T-cell function, as evidenced by rejection of allogeneic skin grafts. In both thymectomized and unthymectomized mice subjected to whole-body irradiation, the lung-colony-forming efficiency was high 1 day after irradiation, declined to a minimum at 7 days, and thereafter increased again, unless the animals were held in a pathogen-free environment. Reconstitution of T-cell-depleted mice with thymocytes and/or a thymic lobe graft tended to increase further, rather than reduce, lung-colony-forming efficiency. Induction of profound lymphopenia, by irradiation of the whole body except the thorax, did not significantly increase lung colony yields. These studies show that the lung colony assay is not a reliable method of assessing T-cell function in irradiated mice.**

THE lung colony assay, introduced by Hill and Bush (1969), is an economical and popular method of *in vivo* assay of tumour-cell clonogenicity. The assay is performed by injecting cells from disaggregated tumours i.v. into assay animals. After an appropriate interval (usually 2–3 weeks) the animals are killed, and the number of macroscopic tumour nodules on the surface of the lungs is counted. Within certain limits, the number of colonies is proportional to the number of reproductively viable (clonogenic) cells in the inoculum. The characteristically low efficiency of the assay (number of lung colonies formed for a given number of injected cells) is increased, *inter alia*, by irradiation of the lungs prior to injection of tumour cells (Dao and Yogo, 1967; Withers and Milas, 1973; Brown, 1973; van den Brenk *et al.*, 1973; Thompson, 1974). The mechanism by which lung-colony-forming efficiency (CFE) is increased by irradiation is unknown, but the effect is found whatever the tumour-cell immunogenicity. Using a demonstrably immunogenic murine fibrosarcoma, we found, not unexpectedly, that lung CFE was enhanced more by equivalent doses of whole-body irradiation (WBI) than local thoracic irradiation (LTI). However, our initial presumption that this increase was simply due to abrogation of the specific T-cell-dependent antitumour immune response elicited by this tumour (Milas *et al.*, 1975) proved to be unjustified. Our data show that interpretation of results of the lung colony assay in mice deprived of T cells by the classical technique of thymectomy, irradiation and syngeneic bone-marrow reconstitution (TIR) can be extremely difficult, and that

* Present Address: University of Edinburgh, Department of Bacteriology, University Medical School, Edinburgh, Scotland.

Correspondence to: L. J. Peters, M.D., Section of Experimental Radiotherapy, The University of Texas System Cancer Center, M. D. Anderson Hospital and Tumor Institute, 6723 Bertner Avenue, Houston, Texas 77030.

the assay may yield results which are not indicative of T-cell function.

### MATERIALS AND METHODS

*Mice.*—C3Hf/Bu mice of both sexes were used. These mice were bred and maintained in the specific-pathogen-free animal colony of M. D. Anderson Hospital, Section of Experimental Radiotherapy laboratory. However, to gain access to an X-ray machine suitable for zonal irradiations, mice for most experiments were removed from the closed animal colony and thereafter housed in an open environment, 5 to a cage. Within each experiment, mice of the same sex aged 10–12 weeks (except as noted below) were used.

*Tumour.*—The tumour used in these experiments is a methylcholanthrene-induced fibrosarcoma (FSa) which is immunogenic in its syngeneic hosts (Suit and Kastelan, 1970). Source material for the experiments described in this paper was derived from injection of fourth-generation isotransplants. The method used for preparing single-cell suspensions from this tumour has been described previously (Milas *et al.*, 1974).

*Irradiations.*—A Phillips X-ray therapy machine was used at 250 kVp, and 15 mA with 0·5 mm Cu added filtration. The HVL of the beam was 1·3 mm Cu and the dose rate, 91 rad/min at 50 cm. For irradiations involving shielding, mice were anaesthetized with Nembutal 60 mg/kg and specially fabricated shields of 3-mm-thick lead were placed to expose only the required body segments. In one experiment, mice received WBI with a caesium $\gamma$-ray source to a dose of 1000 rad, which is biologically equivalent to 900 rad 250 kVp X-rays. WBI mice were protected from bone-marrow death by reconstitution with syngeneic bone marrow either immediately after exposure or, in one experiment, 4 days later.

*Production of chronically T-cell-depleted mice.*—Mice were thymectomized at 6 weeks of age and 1 week later were given 900 rad WBI, followed immediately by an i.v. injection of $4\times10^6$ syngeneic bone-marrow cells. Such mice are designated TIR (thymectomized, irradiated, reconstituted). Age-matched controls received the same treatment except that thymectomy was omitted and are designated IR. These mice were used in experiments 2–3 months after preparation.

*Lung colony assay.*—Single-cell suspensions of FSa in Hsu's medium were prepared. The required number of viable tumour cells was injected i.v. in a volume of 0·25 ml. For scoring of lung CFE, mice were killed 14 days after tumour-cell injection, and their lungs were removed and fixed in Bouin's solution and the macroscopic surface colonies were counted.

### RESULTS

### *Effects of regional irradiation on lung CFE*

Whole body irradiation (WBI) with 900 rad 250 kVp X-rays given 1 day before tumour-cell injection increases the efficiency of the lung colony assay in our system about 11-fold. A corresponding dose delivered to the thorax only (LTI) increases the lung CFE 3- to 4-fold. Table I shows that no specific body region is critically responsible for this disparity, but rather that the yield of colonies increases progressively with the volume irradiated *provided* the thorax is included. Interestingly, we found that, if the thorax was shielded while the remainder of the body was given 900 rad, the yield of lung colonies was not significantly increased over controls, in spite of profound lymphopenia produced by the radiation exposure: (the total circulating mononuclear leucocyte count in irradiated mice at the time of tumour-cell injection was $<200/$

TABLE I.—*Number of Lung Colonies from* $10^4$ *C3H Fibrosarcoma Cells Injected i.v. 24 h after Irradiation* (900 *rad* 250 *kVp X-rays HVL*=1·3 *mm Cu*) (10 *Mice/Group*)

| Volume of mouse irradiated | Lung colonies ±s.e. mean |
|---|---|
| Nil | 8·2±1·1 |
| Thorax only | 27·9±3·5 |
| Whole body except thorax | 10·8±1·6 |
| Thorax+head | 31·3+4·3 |
| Thorax+abdomen | 62·4±5·6 |
| Thorax+head+abdomen | 68·1±8·3 |
| Whole body* | 90·2±5·9 |

* Animals were "rescued" with $4\times10^6$ bone marrow cells injected i.v. 4 days after irradiation. Reconstitution was delayed to prevent a possible effect on tumour-cell lodgement in the lungs by a closely-timed i.v. injection of marrow cells.

mm³, compared with control counts of ~6500/mm³).

*Recovery from effects of WBI—role of T cells*

If the extra effect of WBI compared with LTI were due to T-cell depletion, recovery from the effect of WBI should be retarded in mice subjected to thymectomy before WBI. Fig. I shows that this was not the case. In two separate pairs of experiments we noted essentially identical recovery patterns in WBI mice that had or had not been thymectomized before irradiation. Significantly, both groups of mice showed a secondary increase in the yield of lung colonies from cells injected more than 7 days after irradiation. This is in contrast to the situation in LTI mice, where the lung CFE declines close to control values by 4 days after irradiation, and then remains constant for at least 28 days (data not plotted).

FIG. 1.—Number of lung colonies from $10^4$ C3H fibrosarcoma cells at various times after 900 rad (250 kVp X-rays) whole-body irradiation. Solid symbols, mice thymectomized one week before whole body irradiation; open symbols, unthymectomized irradiated controls. In the experiment designated by square symbols, the number of colonies in untreated control mice was inexplicably low, which accounts for the vertical displacement of this pair of curves. However, it is apparent that the time course of CFE after WBI is not influenced by thymectomy. Errors represent s.e. mean of 8–10 mice per datum point.

To assess further the effect on lung CFE of acute T-cell depletion by WBI, we reconstituted mice immediately after irradiation with $10^8$ normal thymocytes (along with $10^7$ syngeneic bone marrow cells) and 7 days later injected $10^4$ FSa cells i.v. These mice developed a mean of $13\cdot3 \pm 2\cdot9$ colonies, compared with $8\cdot6 \pm 1\cdot5$ in mice that were not reconstituted with thymocytes.

*Lung CFE in mice chronically depleted of T cells*

Mice were rendered T-cell deficient by the technique of thymectomy, irradiation and syngeneic bone-marrow reconstitution (TIR) while age-matched controls received irradiation and bone-marrow reconstitution (IR) only. Two to three months later, the T-cell function of these mice was assessed by their ability to

TABLE II.—*Results of Lung Colony Assays from $10^4$ or $10^5$ C3H Fibrosarcoma Cells Injected i.v. 2–3 Months after WBI.* (8 *Mice/Group*)

| | Tumour cells injected | | |
|---|---|---|---|
| | | $10^5$ | |
| Condition of mice | Lung colonies ±s.e. | Lung colonies | Lung weights (mg)§ mean ±s.e. |
| Intact | 11·9±2·8 | — | |
| TIR* | 109·7±4·1 | 300–400 | 797±57 |
| IR† | 84·8±9·6 | — | |
| TIR+T cells‡ | — | Confluent (>400) | 1124±30 |

*Mice were thymectomized when 6 weeks old, and one week later received 900 rad whole-body irradiation followed by syngeneic bone-marrow reconstitution (4 × $10^6$ nucleated cells).

†Age-matched mice received identical treatment, except that thymectomy was omitted.

‡TIR mice were injected i.v. with 5 × $10^6$ syngeneic thymocytes, and a normal thymic lobe was implanted 5 days before tumour-cell challenge.

§Age-matched normal range 188±7 mg.

reject allogeneic BALB/c skin grafts. Whereas TIR mice accepted the foreign skin grafts indefinitely, IR mice rejected them in 12–14 days, indicating that their T-cell function had recovered. The results of lung colony assays in TIR and IR mice are presented in Table II. These show that IR mice developed almost as many colonies as TIR mice, in spite of having recovered their T-cell competence. In both cases, the yield of lung colonies was significantly higher than in unirradiated controls. Data in Table II also show that grafting of syngeneic thymic tissue to TIR recipients 5 days before tumour-cell injection was ineffective in reducing the yield of lung colonies in such mice. On the contrary, the yield of colonies and the lung weights were further increased.

*Possible influence of pulmonary infection in the lung colony assay*

The reason for the secondary sustained increase in yield of lung colonies in mice subjected to WBI is uncertain. Such an increase is not seen in LTI mice. One possibility we entertained was that WBI predisposed animals to secondary pulmonary infection, which increased the efficiency of lung colony formation. Evidence supporting this theory comes from the following experiment: WBI mice were held in either a pathogen-free environment or an unprotected environment, for varying periods between WBI and the injection of tumours cells. The results presented in Fig. 2 show that the secondary increase in lung CFE did not occur in mice kept in the pathogen-free environment.

FIG. 2.—Relative number of lung colonies at different times after lethal WBI (900 rad, 250 kVp X-rays or 1000 rad $^{137}$Cs $\gamma$-rays) according to the habitat of the mice after irradiation. ● ○, mice kept in "conventional" conditions; △, mice kept in "pathogen-free" environment. Colony numbers normalized to 50 on Day 7.

### DISCUSSION

These experiments show that certain pitfalls exist in the interpretation of the lung colony assay in whole-body-irradiated animals. The technique of TIR (Miller, Doak and Cross, 1963) has become a standard method of producing long-lasting T-cell depletion. However, it is wrong to assume that an increased yield of lung colonies in TIR mice is directly ascribable to T-cell depletion. We have found that unthymectomized mice subjected to IR 2–3 months before tumour-cell injection have almost the same lung CFE as TIR mice, in spite of the recovery of T-cell function in IR mice, as assessed by allogeneic skin graft rejection. Also, we found that engraftment of normal thymic tissue into TIR mice resulted in a further *increase* in the yield of lung colonies. A similar enhancement of lung CFE was noted when reconstitution with $10^8$ thymocytes was performed immediately after WBI, suggesting, perhaps, that restoration of a weak immune response to the tumour was beneficial to tumour growth (Prehn, 1972). The failure of T-cell reconstitution to reduce the yield of lung colonies in irradiated mice could be explained by a suboptimal number of reconstituting cells, or by a relative preponderance of suppressor cells, in the reconstituting T-cell populations. However, these explanations cannot account for the sustained high lung CFE in mice whose T-cell function had recovered spontaneously, and we would caution against the use of the lung colony assay in TIR mice unless the appropriate IR, rather than the customary sham-thymectomized controls are used.

All investigators who have studied the effects of LTI on lung CFE in mice have found that the effect decays rapidly after irradiation and, in most cases, no enhancement of lung CFE was reported later than 1–4 weeks after irradiation. In one instance, however, a delayed effect was seen 3 1/2 months after 2000 rad to one hemithorax (Thompson, 1974). In contrast to this usually rapid recovery following LTI, we have found that after the same dose of WBI, an initial fall in lung CFE is followed by a secondary rise 7–21 days after exposure, when animals were housed in an open environment. The secondary increase was not seen, however, when mice were kept in a pathogen-free environment. This finding suggests that infection may play a role in the genesis of the secondary increase we noted, and adds another complicating factor in the use of the lung colony assay in WBI animals.

Finally, it is of interest that the induction of profound lymphopenia by irradiation of the whole body except thorax did not significantly increase lung CFE, even though the tumour we used exerts strong immunogenicity. This is consistent with the view that the development of a specific antitumour immune response has little influence on the yield of lung colonies after i.v. injection of tumour cells, because the fate of the great majority of injected tumour cells is determined within 24 h of injection, long before a specific immune response could be mounted. For example, studies with $^{125}$IUdR-labelled FSa cells (to be reported elsewhere) show that following injection of $10^4$ tumour cells into intact animals, only 0·5% (50 cells) remain in the lungs 24 h later.

Animals used in this study were maintained in facilities approved by the American Association for Accreditation of Laboratory Animal Care, and in accordance with the current United States Department of Agriculture and Department of Health, Education and Welfare, National Institues of Health regulations and standards.

### REFERENCES

BROWN, J. M. (1973) The Effect of Lung Irradiation on the Incidence of Pulmonary Metastases in Mice. *Br. J. Radiol.*, **46**, 613.

DAO, T. L. & YOGO, H. (1967) Enhancement of Pulmonary Metastases by X-irradiation in Rats Bearing Mammary Carcinoma. *Cancer, N.Y.*, **20**, 2020.

HILL, R. P. & BUSH, R. S. (1969) A Lung Colony Assay to Determine the Radiosensitivity of the Cells of a Solid Tumour. *Int. J. Radiat. Biol.*, **15**, 435.

MILAS, L., HUNTER, N., MASON, K. & WITHERS, H. R. (1974) Immunological Resistance to Pulmonary Metastases in C3Hf/Bu Mice Bearing Syngeneic Fibrosarcoma of Different Sizes. *Cancer Res.*, **34**, 61.

MILAS, L., KOGELNIK, H. D., BASIC, I., MASON, K., HUNTER, N. & WITHERS, H. R. (1975) Combination of *C. parvum* and Specific Immunization Against Artificial Pulmonary Metastases in Mice. *Int. J. Cancer*, **16**, 738.

MILLER, J. F. A. P., DOAK, S. M. A. & CROSS, A. M. (1963) Role of Thymus in Recovery of the Immune Mechanism in the Irradiated Adult Mouse. *Proc. Soc. exp. Biol. Med.*, **112**, 785.

PREHN, R. T. (1972) The Immune Reaction as a Stimulator of Tumor Growth. *Science, N.Y.*, **176**, 170.

SUIT, H. D. & KASTELAN, A. (1970) Immunological Status of Host and Response of a Methylcholanthrene-induced Sarcoma to Local X-Irradiation. *Cancer, N.Y.*, **26**, 232.

THOMPSON, S. C. (1974) Tumour Colony Growth in the Irradiated Mouse Lung. *Br. J. Cancer*, **30**, 337.

VAN DEN BRENK, H. A. S., BURCH, W. M., ORTON, C. & SHARPINGTON, C. (1973) Stimulation of Clonogenic Growth of Tumour Cells and Metastasis in the Lungs by Local X-Irradiation. *Br. J. Cancer*, **27**, 291.

WITHERS, H. R. & MILAS, L. (1973) Influence of Preirradiation of Lung on Development of Artificial Pulmonary Metastases of Fibrosarcoma in Mice. *Cancer Res.*, **33**, 1931.